# Neuropathology of supercentenarians - four autopsy case studies

**DOI:** 10.1186/s40478-016-0368-6

**Published:** 2016-09-02

**Authors:** Masaki Takao, Nobuyoshi Hirose, Yasumichi Arai, Ban Mihara, Masaru Mimura

**Affiliations:** 1 Department of Neurology, Saitama Medical University International Medical Center, Yamane, 1397-1 Yamane, Hidaka, Saitama 350-1298 Japan; 2 Center for Supercentenarian Medical Research, School of Medicine, Keio University, 35 Shinanomachi, Shinjuku-ku, Tokyo, 160-8582 Japan; 3 Department of Neurology and Brain Bank, Mihara Memorial Hospital, 366 Ohtemachi, Isesaki, Gunma 372-0006 Japan; 4 Department of Neuropsychiatry, School of Medicine, Keio University, 35 Shinanomachi, Shinjuku-ku, Tokyo, 160-8582 Japan

**Keywords:** Aging, Supercentenarian, Neuropathology, Amyloid-beta, Tau, TDP-43

## Abstract

Supercentenarians (aged 110 years old or more) are extremely rare in the world population (the number of living supercentenarians is estimated as 47 in the world), and details about their neuropathological information are limited. Based on previous studies, centenarians (aged 100–109 years old) exhibit several types of neuropathological changes, such as Alzheimer’s disease and Lewy body disease pathology, primary age-related tauopathy, TDP-43 pathology, and hippocampal sclerosis. In the present study, we provide results from neuropathological analyses of four supercentenarian autopsy cases using conventional and immunohistochemical analysis for neurodegenerative disorders. In particular, we focused on the pathology of Alzheimer’s disease and Lewy body disease, as well as the status of hippocampal sclerosis, TDP-43 pathology, aging-related tau astrogliopathy, and cerebrovascular diseases. Three cases were characterized as an “intermediate” level of Alzheimer’s disease changes (NIA-AA guideline) and one was characterized as primary age-related tauopathy. TDP-43 deposits were present in the hippocampus in two cases. Neither Lewy body pathology nor hippocampal sclerosis was observed. Aging-related tau astrogliopathy was consistently observed, particularly in the basal forebrain. Small vessel diseases were also present, but they were relatively mild for cerebral amyloid-beta angiopathy and arteriolosclerosis. Although our study involved a small number of cases, the results provide a better understanding about human longevity. Neuropathological alterations associated with aging were mild to moderate in the supercentenarian brain, suggesting that these individuals might have some neuroprotective factors against aging. Future prospective studies and extensive molecular analyses are needed to determine the mechanisms of human longevity.

## Introduction

Increased human longevity is a goal in many parts of the world. Although it is difficult to clearly define “successful aging,” aging without any severe diseases or disabilities is ideal. Among the many factors associated with a decreased ability to function during human aging, brain disease is an important factor. According to epidemiological data, the number of centenarians (aged 100 or more) in the world was 451,000 in 2015 (http://esa.un.org/unpd/wpp/Download/Standard/Population; World Population Prospects, 2015 revision). Additionally, this number could increase to 3,678,000 by 2050 (http://esa.un.org/unpd/wpp/Download/Standard/Population; World Population Prospects, 2015 revision). Neuropathological analyses of centenarian brains remain limited. From a Japanese cohort, centenarian brains exhibited Alzheimer’s disease (AD) and cerebrovascular disease (CVD), as well as cases with no definite pathological alterations [1]. Based on our introductory analysis of 58 centenarian brains (mean age 101.5 ± 1.7 y), a high likelihood of intermediate AD pathology (NIA Reagan criteria) was observed in 15 cases (25.8 %) and a low likelihood of sparse neuritic plaques and stage I/II of Braak staging of neurofibrillary tangles was observed in 8 cases (13.7 %). Additionally, 21 cases (36.2 %) were characterized as primary age-related tauopathy (PART), which was recently recognized as a neuropathological condition of the aging brain. Recent published neuropathological data from three cohorts in the US and UK showed that AD pathology is not common [2]. Hippocampal sclerosis-related aging and Lewy body pathology were reported, and arteriolosclerosis was shown to be possibly associated with hippocampus sclerosis-related aging [2, 3]. Therefore, those centenarian cases may exhibit high proportions of AD pathology, as well as non-AD pathology. However, the average age of individuals analyzed in those studies was close to 100 years. In autosomal-dominant cases of AD, the onset of amyloid-beta deposition might be 15 years prior to the onset of clinical symptoms [4]. Therefore, centenarians close to 100 years of age with AD pathology might represent individuals who developed pathological AD changes between 80 and 90 years of age.

Compared with centenarians, supercentenarians (aged 110 years old or more) are extremely rare in the human race. According to a study by the “Gerontology Research Group” in 2016, the number of living supercentenarians is estimated as 47 in the world (http://supercentenarian-research-foundation.org/TableE.aspx, accessed July 14, 2016). However, it is difficult to estimate the actual number of worldwide supercentenarians, because some individuals lack a birth certificate or, depending on the country of origin, it might be difficult to obtain a reliable birth certificate. Therefore, systematic neuropathological analysis of supercentenarian brains remains difficult. We have attempted to locate supercentenarians and communicate with their families to obtain brain samples for pathological analyses. Clarification of the neuropathological conditions of these exceptional humans might provide a better understanding of the pathomechanisms involved in human longevity.

A previous clinical and neuropathological study of a 115-year-old woman from the Netherlands [5] reported well-preserved cognitive function, with only mild aging alterations. In the present study, we provide results from neuropathological analyses of four supercentenarian autopsy cases using conventional and immunohistochemical analysis for neurodegenerative disorders. In particular, we focused on the pathology of AD and Lewy body disease, as well as the status of hippocampal sclerosis, TDP-43 pathology, aging-related tau astrogliopathy (ARTAG), and CVD.

## Materials and methods

### Clinical information

All individuals were contacted by one (NH) of the authors prior to death. At that time, clinical conditions and some medical anamnesis were obtained from each individual and their relatives, as well as their caregivers. Blood samples were obtained for future analyses. To confirm the age of individuals, the date of birth was determined using the family register. Apolipoprotein E (APOE) genotyping was also determined.

### Autopsy

Because the individuals were placed in elderly care facilities, the bodies were transferred from the facilities to our brain bank by hospital ambulance following death. At the time of autopsy, fresh brain tissue was dissected at the mid-sagittal line. The right cerebrum, cerebellum, and brainstem were immediately frozen by dry ice and stored at −80 °C for future studies. The left hemisphere of the brain was fixed in 20 % neutral-buffered formalin (Wako, Osaka, Japan) for neuropathological analysis.

### Neuropathological studies

Neuropathological studies were performed on postmortem tissue from four individuals (Table 1). All cases were registered with our Brain Bank and neuropathologically analyzed according to the following protocols. Samples were dissected from coronal slices of fixed brains from the following regions: superior and middle frontal gyri, anterior cingulate gyrus, superior and middle temporal gyri, motor and sensory cortices, insular cortex, calcarine cortex, amygdala, hippocampus, subiculum, parahippocampal gyrus, caudate nucleus, putamen, globus pallidus, thalamus, cerebellum, midbrain, pons, and medulla [6, 7]. Blocks were dehydrated in alcohol gradients, cleared in xylene, and embedded in paraffin. The brain tissues were cut into 6-μm-thick sections. The sections were stained with hematoxylin and eosin (HE), Klüver-Barrera for myelin, and modified Gallyas-Braak silver staining for fibrils. For immunohistochemical studies, monoclonal antibodies specific to amyloid Aβ (11–28) (12B8, 1:100, IBL, Gunma, Japan), phospho-tau (AT8, 1:3000, Innogenetics, Ghent, Belgium), 4-repeat isoform tau (RD4, 1:500, 1E1/A6, Merck Millipore, Darmstadt, Germany), 3-repeat isoform tau (RD3, 1:500, 8E6/C11, Merck Millipore), phosphorylatedα-synuclein (1:7000, pSyn#64, monoclonal, Wako), and phosph-TDP-43 (s409/410, 1:7000, COSMO BIO, Tokyo, Japan) were used. The sections were processed using a Ventana Discovery automated immunostainer (Roche, Basel, Switzerland), and the sections were counterstained with hematoxylin.

**Table 1:** Patient demographics and summary of neuropathological analyses

	Case 1 (111 yo)	Case 2 (111 yo)	Case 3 (114 yo)	Case 4 (110 yo)
Sex	Female	Female	Female	Female
Past medical history	HT-, DM-	HT-, DM-	HT-, DM-	HT+, DM-
Clinical condition before death	Clear communication, wheel chair	No dementia	Clear communication, wheel chair	Almost clear communication, wheel chair
Barthel index CDR MMSE	10 0.5 NA due to refusal Age 106	60 NA 15 Age 106	90 0 22 Age 108 MMSE was 18 at age 113 and 114	20 NA NA Age 109
Cause of death	Heart failure	Renal failure	Senility	Sepsis
APOE	2/3	2/3	3/3	3/3
Brain weight (fresh)	460 (left hemisphere)	925	1,015	1,115
Atrophy	F, T	F, T	F, T	T
A-beta, Thal phase	3 (A2)	3 (A2)	1 (A1)	2 (A1)
NFT stage (AT8) (Braak)	III (B2)	IV (B2)	III (B2)	IV (B2)
Neuritic plaques (CERAD)	Moderate (C2)	Moderate (C2)	Sparse (C1)	Moderate (C2)
CAA	None	None	None	Mild
AD pathological changes (NIA-Reagan)	Intermediate	Intermediate	Unclassified	Intermediate
AD pathological changes (NIA-AA)	Intermediate	Intermediate	Low PART possible	Intermediate
ARTAG	Subpial, subependymal, gray matter, white matter, perivascular	Subpial, gray matter, white matter	Subpial, gray matter, white matter	Subpial, subependymal, gray matter, perivascular
Arteriolosclerosis	Mild to moderate	Mild to moderate	Mild to moderate	Mild to moderate
White matter rarefaction	Moderate	Mild	Mild	Moderate
État criblé (basal ganglia and thalamus)	Moderate	Severe	Severe	Moderate
Vascular brain injury	Multiple cortical infarcts	None	None	None
Alpha-synuclein pathology	None	None	None	None
TDP-43 pathology	Present, subiculum, PHG	Present, subiculum, PHG	Uncus, sparse	Uncus, sparse
Hippocampal sclerosis	None	None	None	None
Hirano bodies/GVD (Hippocampus)	Present/present	Present/present	Present/present	Present/present

Abbreviations: *ARTAG* aging-related tau astrogliopathy, *CAA* cerebral amyloid angiopathy, *CDR* Clinical Dementia Rating, *CERAD* Consortium to Establish a Registry for Alzheimer’s Disease, *DM* diabetes mellitus, *F* frontal lobe, *GVD* granulovaculaor degeneration, *HT* hypertension, *MMSE* Mini-Mental State Examination, *NA* not available, *NIA* National Institute on Aging, *NIA-AA* NIA-Alzheimer’s Association, *T* temporal lobe, *yo* years old

For neuropathological diagnosis of Alzheimer’s disease, we used the Consortium to Establish a Registry for Alzheimer’s Disease (CERAD) methodologies, widely applied to make a diagnosis of Alzheimer’s disease, as a guide for analyzing the frequency of neuritic plaques [8]. Braak staging of neurofibrillary tangle formation and Thal phase of amyloid beta were also used [9–11]. Eventually, National Institute on Aging (NIA)-Reagan criteria (1997) and NIA-Alzheimer’s Association (NIA-AA) guidelines (2012) were applied for determining the level of AD pathological change [12, 13]. Those neuropathological strategies also allowed us to identify whether each case corresponded with PART criteria.

The ARTAG type and anatomical areas were analyzed in all sections according to a recently published harmonized evaluation strategy using AT8 immunohistochemistry [14]. First, AT8 immunoreactive thorn-shaped astrocytes (TSA) and fine granular immunoreactivity (GFA) were identified. Second, the type, major anatomical involvement, severity, and detailed regional distribution were determined [14]. TDP-43 immunoreactivity was classified as neuronal cytoplasmic (NCI), neuronal nuclear (NNI), and glial cytoplasmic (GCI) immunoreactive deposits, as well as neurites.

Cerebral amyloid angiopathy and arteriolosclerosis, as well as white matter rarefaction and état criblé, were assessed using a subjective scale of none, mild, moderate or severe.

We obtained written informed consent from the relatives of the deceased for autopsy and further neuropathological analysis, and all subjects were registered with our brain bank for future research. The brain bank was approved by the Ethics Committee of Mihara Memorial Hospital for neuropathological analysis.

## Results

### Clinical information

All four supercentenarian individuals were female. Because they were placed in elderly care facilities, the individuals did not receive extensive medical treatment during the end stages of life. It was difficult to determine whether they were demented at the time of interview. Case 1 was able to communicate and move her wheelchair herself. In Case 2, no apparent dementia symptoms were reported by the nursing staff. Cases 3 and 4 were able to communicate with other people and manipulate their wheel chairs. In some individuals, Barthel index, clinical dementia rating (CDR) scale and Mini-Mental State Examination (MMSE) were evaluated (Table 1). The clinical characteristics are summarized in Table 1.

### Neuropathology

Because brain pathology of supercentenarians has not been previously well described, we report descriptive neuropathological findings from each case. The summary of neuropathological findings is shown in Table 1.

### Case 1

#### Gross neuropathology

**Fig. 1:**
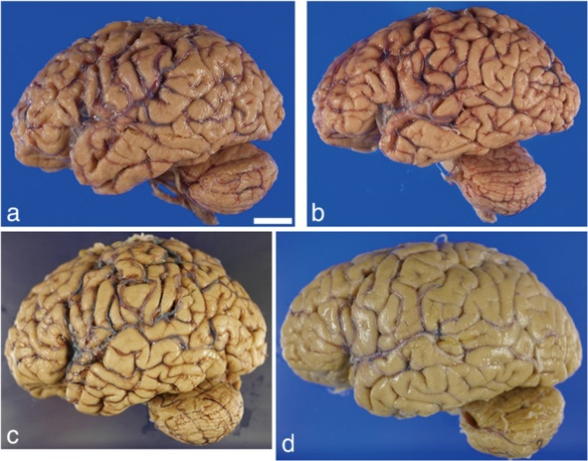
Photomicrographs of fixed left-brain hemispheres from four supercentenarians. Mild atrophy is present in the frontal and/or temporal lobes. **a** Case 1, (**b**) Case 2, (**c**) Case 3, (**d**) Case 4. Bar = 2 cm

**Fig. 2:**
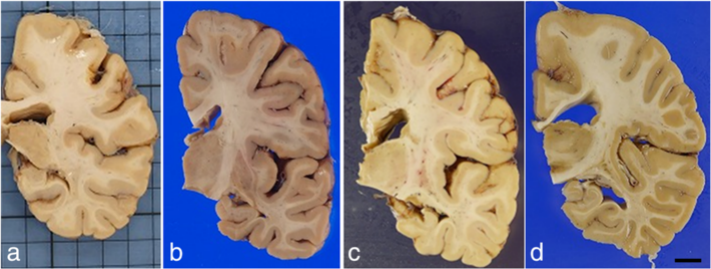
Coronal sections at the basal ganglia and hippocampus level. Cortical ribbons and cerebral white matter are well-preserved. Small cortical infarct in Case 1 (**a**). **a** Case 1, (**b**) Case 2, (**c**) Case 3, (**d**) Case 4. Bar = 1 cm

The fresh brain weight of the left hemisphere was 460 grams. Unfortunately, the right cerebral hemisphere (frozen side) was not weighed at the time of autopsy. After formalin fixation, there was mild atrophy in the frontal and temporal lobes (Figs. 1a and 2a). Atherosclerosis was mild in the major cerebral arteries. No atheromatous plaques were seen in the leptomeningeal vessels. On the coronal sections, a mild enlargement was present at the posterior part of lateral ventricle. No atrophy was seen in the cerebellum. The substantia nigra and locus coeruleus were well pigmented.

#### Microscopic neuropathology

**Fig. 3:**
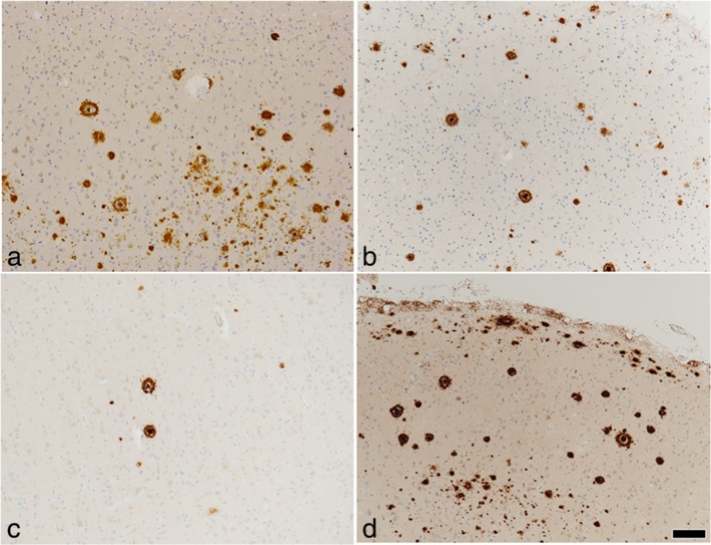
Photomicrographs of the temporal cortices from four cases. Immunohistochemistry using monoclonal antibody specific to Aβ (11–28). **a** Case 1, (**b**) Case 2, (**c**) Case 3, (**d**) Case 4. Bar = 100 μm

**Fig. 4:**
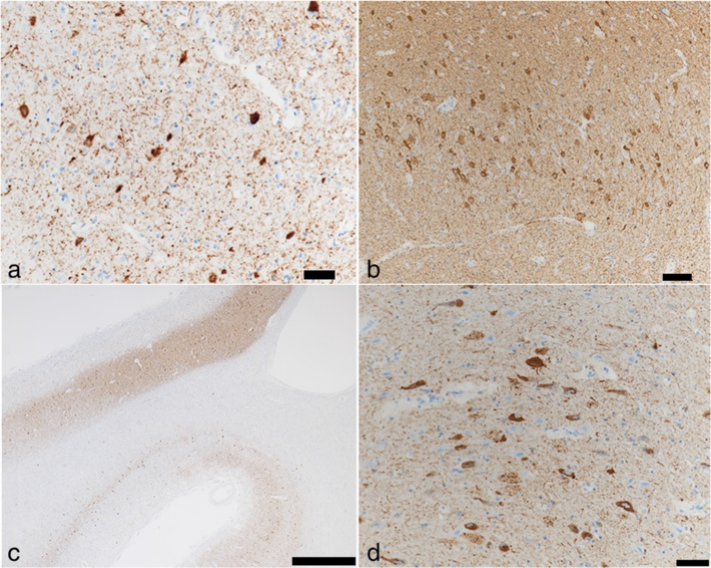
Photomicrographs of hippocampus and parahippocampus from four cases. Immunohistochemistry using monoclonal antibody specific to p-tau (AT8). **a** Case 1, (**b**) Case 2, (**c**) Case 3, (**d**) Case 4. Bar = 50 μm

**Fig. 5:**
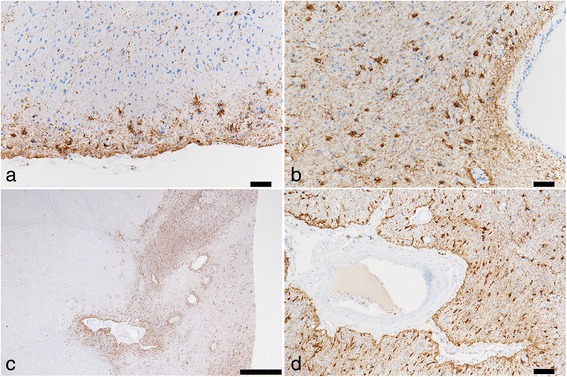
ARTAG of Case 1. ARTAG (thorn-shaped astrocytes) is present in the subpial areas of inferior temporal gyrus (**a**), white matter close to the lateral ventricle of the hippocampus (**b**), and perivascular area of the basal forebrain (**c**, **d**). Immunohistochemistry using monoclonal antibody specific to p-tau (AT8). Bar = 50 μm (**a**, **b**, **d**). Bar = 1000 μm (**c**)

**Fig. 6:**
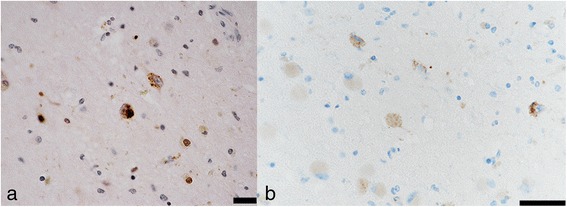
TDP-43 pathology in the subiculum (**a**) and basal forebrain (**b**) from Case 1. Neuronal cytoplasmic inclusions and neurites are visible. TDP-43 immunohistochemistry. Bar = 100 μm (**a**). Bar = 50 μm (**b**)

**Fig. 7:**
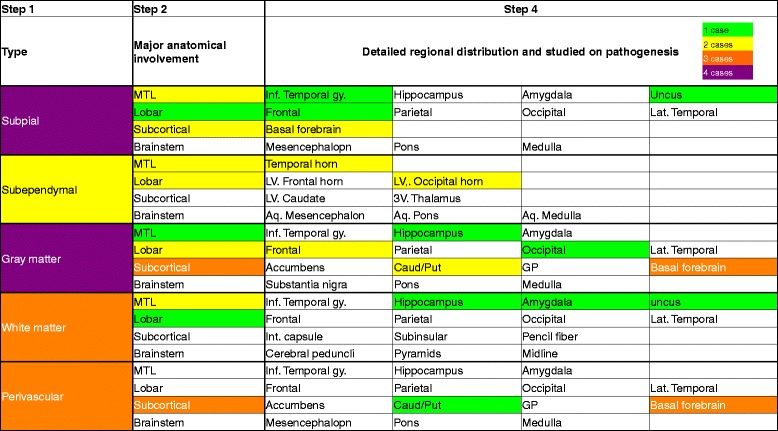
Distribution and severity of ARTAG pathology in four cases. The basal forebrain is a relatively commonly affected area

Neuronal loss and gliosis was none to mild in the majority of the cerebral cortex. Neurons in the substantia nigra and locus coeruleus were well preserved. Moderate numbers of Aß-immunoreactive neuritic plaques were observed using CERAD methodology. Aß-immunoreactive parenchymal deposits were characterized as phase 3 using Thal’s methodology. There was also a moderate level of Aß-immunoreactive diffuse plaques observed, although no Aß-immunoreactive cerebral amyloid angiopathy was seen. AT8-immunoreactive NFTs were stage III according to Braak methodology (Figs. 3a and 4a, Table 1). Therefore, Case 1 was assigned an intermediate level of AD pathological changes according to NIA-Reagan and NIA-AA criteria. Alpha-synuclein immunoreactive deposits, as well as AT8-immunoreactive tufted astrocytes and astrocytic plaques, were not observed. All ARTAG types were mainly observed in the medal temporal lobe and subcortical areas. The perivascular ARTAG pattern was particularly present in the basal forebrain (Figs. 5 and 7). No hippocampal sclerosis was observed. TDP-43-immunoreactive NCIs and neuritis were present in the basal forebrain, hippocampus, and subiculum (Fig. 6, Table 2). NNIs and GCIs were also present (Table 2). In the parietal cortex, there was a small area with neuronal loss, with gliosis and hemosiderin-laden macrophages in the cortex. These findings were consistent with an old hemorrhagic infarct. A small vessel occluded by an organizing thrombus was seen in the subarachnoid space immediately adjacent to the infarct. Because there were no atherosclerotic changes in the leptomeningeal vessels, the infarct was considered to be an embolic lesion. Mild to moderate arteriolosclerosis was observed in various areas (Table 3). Mineralization was seen in the small vessel wall in the globus pallidus (Fig. 9a).

**Table 2:** Severity and distribution of TDP-43-immunoreactive deposition

Areas	Case 1				Case 2				Case 3				Case 4			
	NCI	Neurites	NNI	GCI	NCI	Neurites	NNI	GCI	NCI	Neurites	NNI	GCI	NCI	Neurites	NNI	GCI
Basal ganglia	Basal forebrain; +++	Basal forebrain; +	-	+	-	-	-	-	-	-	-	-	-	-	-	-
Temporal lobe	-	-	-	-	-	-	-	-	-	-	-	-	-	-	-	-
Uncus	-	++	-	+	-	-	-	-	-	+	-	-	-	-	-	+/−
Hippocampus-PHG	CA1; +++ Subiculum; +++ Dentate fascia; +/−	CA1; +++ Subiculum; +++	+	+	CA1; ++ subiculum++ Dentate fascia;+/−	CA1; ++ subiculum++	-	+	-	-	-	-	-	-	-	-
Motor cortex	-	-	-	-	-	-	-	-	-	-	-	-	-	-	-	-
Midbrain	-	-	-	-	-	-	-	-	-	-	-	-	-	-	-	-
Medulla	-	-	-	-	-	-	-	-	-	-	-	-	-	-	-	-
Spinal cord	-	-	-	-	-	-	-	-	-	-	-	-	-	-	-	-

Abbreviations: *GCI* glial cytoplasmic inclusions, *NCI* neuronal cytoplasmic inclusion, *NNI* neuronal nuclear inclusion, *PHG* parahippocampal gyrus

-: none

+: mild

++: moderate

+++: severe

**Table 3:** Small vessel disease and white matter rarefaction

	Arteriolosclerosis				White matter rarefaction état criblé (basal ganglia and thalamus)			
	Case 1	Case 2	Case 3	Case 4	Case 1	Case 2	Case 3	Case 4
Frontal	2	2	1	2	2	0	1	2
Temporal	1	2	1	2	2	0	0	2
Parietal	2	1	2	1	2	1	1	1
Occipital	2	1	0	2	2	0	0	1
Hippocampus	1	1	1	1	1	0	1	1
Basal ganglia	2	2	2	2	2	3	3	2
Thalamus	2	2	2	2	2	2	2	2
Cerebellum	2	2	2	2	0	1	0	1

Instead of white matter rarefaction, the severity of état criblé was analyzed in the basal ganglia and thalamus

0: none, 1: mild, 2: moderate, 3: severe

### Case 2

#### Gross neuropathology

The fresh brain from Case 2 weighed 925 grams. After formalin fixation, there was mild atrophy in the frontal lobe and moderate atrophy in the temporal lobes (Fig. 1b). Atherosclerosis was mild in the major cerebral arteries. No atheromatous plaques were seen in the leptomeningeal vessels. On coronal sections, there were no focal lesions except mild enlargement of the lateral ventricle (Fig. 2b). Subcortical nuclei were well preserved without obvious atrophy. No atrophy was seen in the cerebellum. The substantia nigra and locus coeruleus were well pigmented.

#### Microscopic neuropathology

**Fig. 8:**
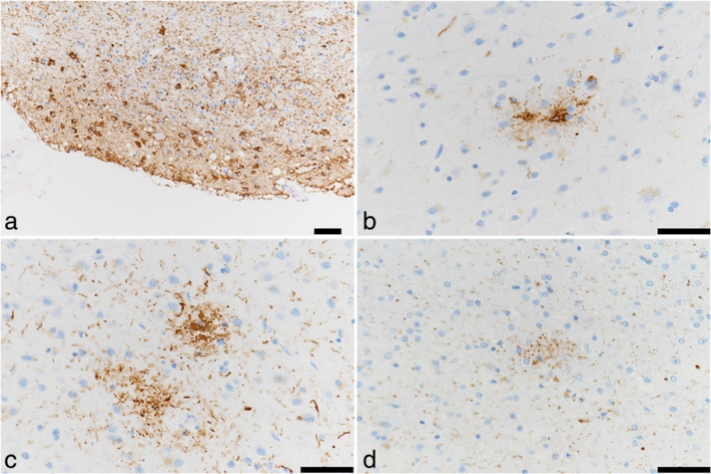
ARTAG of Case 2. ARTAG (thorn-shaped astrocytes) is present in subpial areas of the basal forebrain (**a**). Granular or fuzzy astrocytes (GFA) are seen in the frontal (**b**) and occipital (**c**) cortex. In rare instances, possible GFAs are seen in the temporal white matter (**d**). Immunohistochemistry using monoclonal antibody specific to p-tau (AT8). Bar = 50 μm

**Fig. 9:**
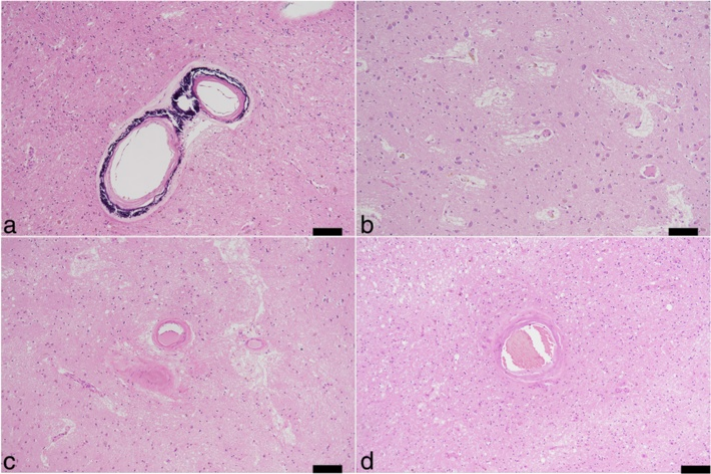
Representative photomicrographs of small-vessel disease (arteriolosclerosis) and état criblé (**a**–**d**). Mineralization is seen in Case 1 (**a**). État criblé is seen in the basal ganglia and thalamus (**b** and **c**). **a** Case 1: globus pallidus, (**b**) Case 2: thalamus, (**c**) Case 3: putamen, (**d**) Case 4: globus pallidus. Bar = 50 μm

Neuronal loss and gliosis was none to mild in the majority of the cerebral cortex. Neurons in the substantia nigra and locus coeruleus were well preserved. Levels of Aß-immunoreactive neuritic plaques and parenchymal deposits were determined moderate by CERAD and phase 3 by Thal’s staging methodology, respectively. There were also sparse levels of Aß-immunoreactive diffuse plaques observed, but no Aß-immunoreactive cerebral amyloid angiopathy was observed. AT8-immunoreactive NFTs were stage IV according to Braak methodology. Therefore, Case 2 was assigned to an intermediate level of AD pathological changes according to NIA-Reagan and NIA-AA criteria (Table 1, Figs. 3b and 4b). Alpha-synuclein immunoreactive deposits and hippocampal sclerosis were not observed, and there were no AT8-immunoreactive tufted astrocytes or astrocytic plaques. ARTAGs were observed in the following regions: 1) subpial/lobar, subcortical/frontal, and basal forebrain, 2) gray matter/subcortical/basal forebrain, and 3) white matter/lobar/lateral temporal (Figs. 7 and 8). The astrocytic tau deposits in the frontal cortex were not associated with Aß deposits. TDP-43-immunoreactive NCIs and neuritis were moderately observed in the hippocampus and subiculum, and GCIs were also seen (Table 2). Mild to moderate arteriolosclerosis was observed (Table 3). The basal ganglia exhibited severe état criblé (Fig. 9b).

### Case 3

#### Gross neuropathology

The fresh brain from Case 3 weighed 1015 grams. After formalin fixation, mild atrophy at the frontal and temporal lobes was observed (Fig. 1c). Atherosclerosis was mild in the major cerebral arteries, and no atheromatous plaques were seen in the leptomeningeal vessels. On coronal sections, there were no focal lesions, but mild enlargement of the lateral ventricle was observed (Fig. 2c). Subcortical nuclei were well preserved without obvious atrophy, and atrophy was not observed in the cerebellum. The substantia nigra and locus coeruleus were well pigmented.

#### Microscopic neuropathology

**Fig. 10:**
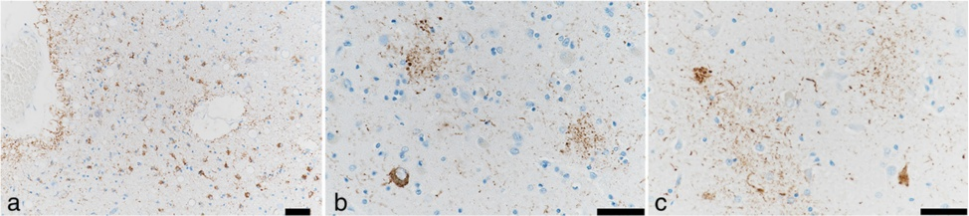
ARTAG of Case 3. ARTAG (thorn-shaped astrocytes) is present in white matter close to the hippocampus (**a**). GFAs are seen in gray matter of the basal forebrain (**b**) and CA4 (**c**). Immunohistochemistry using monoclonal antibody specific to p-tau (AT8). Bar = 50 μm

Neuronal loss and gliosis was none to mild in the majority of the cerebral cortex. Neurons in the substantia nigra and locus coeruleus were well preserved. The level of Aß-immunoreactive neuritic and diffuse plaques was determined to be sparse according to CERAD methodology. Aß-immunoreactive parenchymal deposits were deemed phase 1 using Thal’s methodology. No Aß-immunoreactive cerebral amyloid angiopathy was observed, and AT8-immunoreactive NFTs were stage III according to Braak methodology. Therefore, Case 3 was assigned an unclassified level of AD pathological changes according to NIA-Reagan criteria and low level according to NIA-AA criteria (Table 1, Figs. 3c and 4c). This pathological pattern was consistent with “PART possible” (NFT stage 3, Thal phase 1) [15]. ARTAGs were classified as follows: 1) subpial/MTL/uncus, 2) gray matter/MTL and subcortical/hippocampus and basal forebrain, and 3) white matter/MTL/uncus (Figs. 7 and 10). There were also no AT8-immunoreactive tufted astrocytes or astrocytic plaques, as well as no alpha-synuclein-immunoreactive deposits, and no hippocampal sclerosis was observed. Some TDP-43-immunoreactive neuritis was sparsely seen in the uncus (Table 2). Mild to moderate arteriolosclerosis and état criblé were also observed (Fig. 9c, Table 3).

### Case 4

#### Gross neuropathology

The fresh brain of Case 4 weighed 1115 grams. After formalin fixation, mild atrophy in the temporal lobe was observed (Fig. 1d). Mild atherosclerosis was seen in the major cerebral arteries, but atheromatous plaques were not observed in the leptomeningeal vessels. On coronal sections, focal lesions were not observed, but there was mild enlargement of the lateral ventricle. Although the subcortical nuclei were well preserved, there were some small lacunes in the basal ganglia. No atrophy was seen in the cerebellum, and the substantia nigra and locus coeruleus were well pigmented. A small parenchymal hemorrhage (<3 mm) was present in the middle pontine base.

#### Microscopic neuropathology

**Fig. 11:**
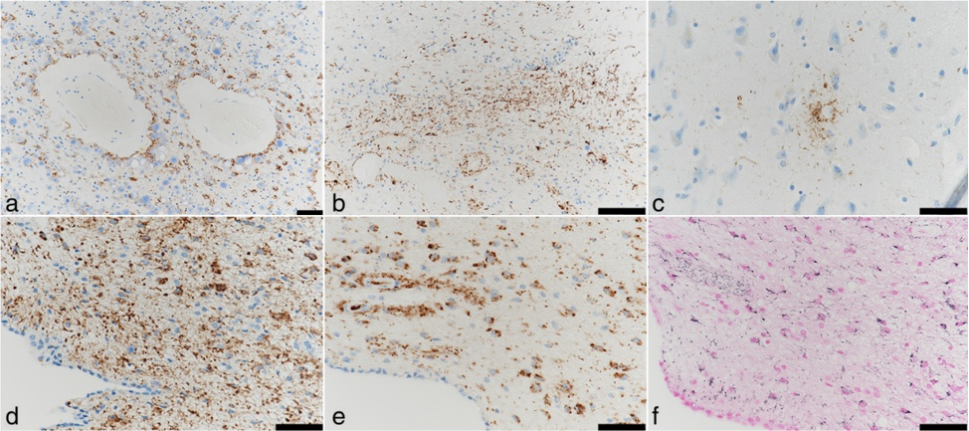
ARTAG of Case 4. ARTAG (thorn-shaped astrocytes) is present in the perivascular area of the basal forebrain (**a**), white matter close to the lateral ventricle of the occipital lobe (**b**), and the lateral ventricle of the medial temporal lobe (**d**, **e**, **f**). GFA is seen in the superior frontal gyrus (**c**). Immunohistochemistry using monoclonal antibody AT8 (**a**–**d**) and RD4 (**e**). Modified Gallyas-Braak stain (**f**). Bar = 50 μm

Neuronal loss and gliosis was none to mild in the majority of the cerebral cortex. Neurons in the substantia nigra and locus coeruleus were well preserved. Aß-immunoreactive neuritic and diffuse plaque scores were moderate and frequent according to CERAD methodology, respectively. Aß-immunoreactive parenchymal deposits were classified as phase 2 according to Thal’s methodology. Aß-immunoreactive cerebral amyloid angiopathy was mildly observed in the parenchymal and leptomeningeal small vessels in the occipital lobe, and AT8-immunoreactive NFTs were considered stage IV using Braak methodology. Therefore, Case 4 was assigned an intermediate level of AD pathological changes according to NIA-Reagan and NIA-AA criteria. There were no AT8-immunoreactive tufted astrocytes or astrocytic plaques. ARTAGs were classified as follows: 1) subpial/subcortical/basal forebrain, 2) subependymal/MTL/temporal lobes, subependymal/lobar/LV of occipital horn, 3) gray matter/lobar/frontal, and 4) perivascular/subcortical/basal forebrain. In all four cases, ARTAG was strongly immunoreactive with AT8 (Figs. 7 and 11). In some instances, ARTAG was also immunoreactive to RD4 antibody, but less depicted by the Gallyas-Braak staining (Fig. 11). This ARTAG immunoreactivity was similar to Cases 1, 2 and 3. Alpha-synuclein-immunoreactive deposits and hippocampal sclerosis were not observed. TDP-43-immunoreactive GCIs were sparsely observed in the uncus (Table 2), and mild to moderate arteriolosclerosis was observed (Fig. 9d, Table 3).

## Discussion

The present study provides neuropathological results from four supercentenarians (≥110 years of age) using conventional and immunohistochemical methods. We emphasize that this novel study is the first unique opportunity to comprehensively determine neuropathological conditions in four supercentenarians. We also introduce NIA diagnostic methodology for Alzheimer’s disease, revealing TDP-43 and ARTAG pathology in those cases. Compared with centenarians, there are currently approximately 50 living supercentenarians in the world (www.grg.org, last updated, July 14, 2016). We believe that supercentenarians are exceptionally distinct human beings, and the study of this cohort is important for understanding the mechanisms of successful aging.

### Clinical information

Because our studies did not perform precise neurological or neuropsychological studies, it was difficult to clinically determine whether the four cases experienced dementia. However, the cases exhibited a certain degree of independence during the last stages of life.

Gender and race might play a role in human longevity. As shown in our results, all four cases were Japanese woman. According to data from the Gerontology Research Group (www.grg.org, last updated, July 14, 2016), most living supercentenarians are women (45/47 individuals). Even in the deceased supercentenarian cohort, the number of men is low, suggesting that it is more difficult for men to reach the supercentenarian age.

Women are considered to be at higher risk of Alzheimer’s disease than men [16, 17]. In previous studies, the rate of dementia increases in women aged > 90 years [18–21]. In a 90+ study, the incidence of dementia increased exponentially with age, reaching 40.7 % (10.9 % men, 38.3 % women) in centenarians [17]. However, gender differences were not observed ≥ 90 years of age [22]. In one study, the incidence of dementia decreased after 97 years of age in women [23].

In centenarian studies, the prevalence of dementia was 27–79 % [24–31]. Conversely, other studies have shown that 37 % of centenarians do not exhibit dementia [32] and may have high cognitive function [24, 33]. This discrepancy between dementia rates may be associated with different methods and samples, as well as selection biases [24]. However, researchers should take care with the relatively low prevalence of dementia in those centenarians as there may be some underestimation [18].

Although four supercentenarians were not clinically considered to exhibit dementia conditions in the present study, they had low MMSE scores. However, visual and hearing impairments pose problems when applying the MMSE test for extremely old individuals [32]. In fact, Case 1 refused to undergo the MMSE test at the time of interview. This suggests that a discrepancy might be present between MMSE and CDR scores in centenarians [32]. Whether MMSE was appropriate for this individual remains unclear. This was further complicated by the fact that Cases 2 and 3 were classified as dementia based on only MMSE scores. Although neuropathological alterations in these four cases likely played a role in cognitive impairment and motor dysfunction, a more precise prospective analysis is needed for extremely old individuals.

Although a previous case report of a female supercentenarian mentioned well-preserved cognitive function [5], the definition of dementia in such extremely aged individuals remains unclear. At this point, we have not validated neuropsychological examinations for the extremely elderly or standard scores for commonly used tests, such as the Mini-Mental State Examination and Frontal Assessment Battery.

Although the Japanese population is considered to have long longevity, there is no strong scientific evidence that longevity is associated with a specific ethnicity or race. In Alzheimer’s disease, many kinds of protective factors have been reported [34]. Although we questioned the relatives about lifestyle and diet factors, we were not able to identify specific factors. The individuals were very physically active and participated in social activities in a positive manner. Future prospective studies of supercentenarians are needed to recruit individuals in their 90s and centenarians.

### Apo E genotype

GWAS studies have shown that the ApoE gene is strongly associated with human longevity [35, 36]. In particular, the ε2 allele is enriched in centenarians, compared with ε4 [37]. In the present study, the ε4 allele was not detected in any of the cases. Although more cases are needed to substantiate our hypothesis, results from the present study suggests that it might be difficult for individuals expressing the ε4 allele to reach a supercentenarian age. Because the ε2 allele is considered to be important for protection of Aβ deposition [38], results from two of our cases suggests that the ε2 allele may affect brain pathology.

### Neuropathology

The neuropathological results from the present study provide unique and imperative findings of brain conditions in supercentenarians. The well-preserved brain shapes and weights in the gross neuropathological findings were surprising. In all cases, there was only mild atrophy in the frontal and/or temporal lobes. Previous results have shown that brain weight is affected by the presence of brain diseases, as well as individual height and gender [39]. Although we could not evaluate the exact height of each individual, the cases were relatively small or of average stature for aged Japanese females. Based on our previous analysis, the mean brain weight of female centenarians (mean age of 101) was 1066 grams (American Association of Neuropathologists, Inc., The 91st Annual Meeting, 2015 Denver, CO. Abstract No. 129). A recent U.S. study also reported that the unfixed brain weight of centenarians is 1000 to 1200 grams [40]. Unfortunately, there is no previous information for the average brain weight of supercentenarians. Nevertheless, we believe that brain weight in these four supercentenarians, in particular Cases 3 and 4, was relatively well-preserved.

Mild atherosclerosis of the major cerebral arteries might be a characteristic feature of supercentenarians. In Case 1, multiple small cortical infarcts with occluded leptomeningeal vessels were observed. Although those infarcts were possibly due to embolic stroke, we were not able to identify the embolism source. Once an individual experiences a major cerebrovascular accident or atherosclerosis of the intracranial cerebral arteries, the clinical prognosis is typically not good [41]. In fact, stroke is one of the most important causes of death and disability worldwide [42]. Results from this study suggest that well-preserved intracranial arteries might be an important element associated with human longevity.

The microscopic findings were also impressive in the four cases. None of them exhibited high-likelihood levels of AD pathology. In one case (Case 3), only low levels of AD changes were observed (Table 1). As stated earlier, age and female sex are generally considered to be risk factors for developing AD [16, 17, 40, 43]. Further, females typically live longer than males, which could increase the risk for developing AD. However, this universal concept has not been extrapolated to supercentenarians, who might have a biological condition that protects against increased tau and Aβ accumulation in the brain.

Primary age-related tauopathy (PART) was recently introduced to describe a pathology commonly observed in the brains of aged individuals [3]. PART is microscopically defined as NFTs with Braak stage ≤ IV (usually III or lower) and Thal Aβ phase 0 (definite) or 1–2 (possible) [3, 15, 44]. In the present study, one case (Case 3) was assigned PART possible. Studies have discussed whether PART is an independent condition or a characteristic of AD [44–46]. At least in our study of PART supercentenarians, there were very few neuritic plaques, and the plaques exhibited an early Thal’s phase of Aß deposition, even at the age of 114 years. It is not currently possible to determine whether extremely aged individuals will also suffer from AD in the future. Further analysis of these brains could provide a better understanding as to how humans can protect against the progression of AD pathology.

ARTAG was recently determined to be a pathological astrocytic condition involving tau accumulation [14]. ARTAGs include not only thorn-shaped astrocytes and granular or fuzzy astrocytes, but also other forms of ARTAG that do not include disease-specific astrocytic tau pathologies, such as tufted astrocytes, astrocytic plaques, ramified astrocytes, or globular astrocytic tau. In our supercentenarians, the medial temporal lobes and basal forebrain were relatively common areas for ARTAGs (Fig. 6), and in those areas, most ARTAGs were thorn-shaped astrocytes. However, ARTAGs were also observed in other brain regions. Nevertheless, the role of ARTAGs remains poorly understood. Because common neurodegenerative pathology is limited in supercentenarians, the presence of ARTAGs could affect clinical and neurological conditions of supercentenarians. However, further analysis is necessary to clarify ARTAG pathology in supercentenarians.

TDP-43 deposition is emphasized in the hippocampus and parahippocampus in aged individuals [2]. Basal forebrain TDP-43 pathology is also strongly associated with hippocampal sclerosis-related aging, which is one of the most important pathological changes indicating dementia [2, 3, 47, 48] and AD pathology [49, 50] in aged individuals. TDP-43 pathology of the hippocampus and parahippocampus was observed in two out of four cases. In Case 1, TDP-43 pathology was only observed in the basal forebrain. Again, hippocampal sclerosis or AD was not diagnosed in the four supercentenarians. Although TDP-43 pathology has been reported in the uncus in normal aging individuals [51], our cases exhibited no severe TDP-43 pathology in the uncus. Additionally, although TDP-43 pathology is considered to spread in a hierarchical manner in individuals with motor neuron disease or frontotemporal lobar degeneration [52, 53], our pathological results did not suggest either of these diseases. Therefore, TDP-43 deposition in our cases could be associated with the aging processes, although the changes were relatively mild. Further studies are needed to determine the pathological changes of TDP-43 in supercentenarian brains.

Arteriolosclerosis was consistently observed, but it was not very severe in the four cases (Table 3). A recent study mentioned that brain arteriolosclerosis could be associated with TDP-43 deposition in the amygdala and limbic system, as well as hippocampal sclerosis [3]. This pathological condition was termed “cerebral age-related TDP-43 pathology and arteriolosclerosis” (CARTS) [3]. Based on this concept, Cases 1 and 2 were at the early clinical stage of CARTS. However, Cases 3 and 4 were at the preclinical stage, and no hippocampal sclerosis was observed in all cases. Therefore, CARTS or hippocampus sclerosis-related aging pathology was a mild condition in the supercentenarians. Because the right cerebral hemispheres in all cases were stored as frozen tissue and were not analyzed, it is important to keep in mind that hippocampus sclerosis-related aging could exist in the right hemisphere.

There are some limitations in the present study. Because all individuals were only visited once or twice by one of the authors, we were not able to obtain detailed clinical information. Also, because neuroimaging studies were not performed, we could not report clinical, neuroradiological, or neuropathological correlations in the present study. We would like to emphasize, however, that it is difficult to obtain autopsies from such extremely old individuals, because they are usually placed in nursing homes or elderly care facilities in Japan.

## Conclusions

Neuropathological alterations associated with aging were relatively mild to moderate in the supercentenarian brain. Considering their extremely old age, the individuals might have some neuroprotective factors against aging. In fact, AD pathological changes of NIA-Reagan and NIA-AA criteria remained low to intermediate in all cases. Future prospective studies and extensive molecular analyses are needed to determine the mechanisms of human longevity. Because we have sufficient frozen tissue from these supercentenarians, we welcome supercentenarian research collaborations.
